# Pathophysiological Mechanisms of Psychosis-Induced Atrial Fibrillation: The Links between Mental Disorder and Arrhythmia

**DOI:** 10.31083/j.rcm2509343

**Published:** 2024-09-24

**Authors:** Pao-Huan Chen, Yu-Hsun Kao, Yi-Jen Chen

**Affiliations:** ^1^Department of Psychiatry, School of Medicine, College of Medicine, Taipei Medical University, 11031 Taipei, Taiwan; ^2^Department of Psychiatry, Taipei Medical University Hospital, 11031 Taipei, Taiwan; ^3^Graduate Institute of Clinical Medicine, College of Medicine, Taipei Medical University, 11031 Taipei, Taiwan; ^4^Department of Medical Education and Research, Wan Fang Hospital, Taipei Medical University, 11696 Taipei, Taiwan; ^5^Division of Cardiology, Department of Internal Medicine, School of Medicine, College of Medicine, Taipei Medical University, 11031 Taipei, Taiwan; ^6^Division of Cardiovascular Medicine, Department of Internal Medicine, Wan Fang Hospital, Taipei Medical University, 11696 Taipei, Taiwan

**Keywords:** atrial fibrillation, autonomic imbalance, serotonin, inflammation, oxidative stress, mitochondrial dysfunction, ion channelopathy, microRNA, schizophrenia, bipolar disorder

## Abstract

Atrial fibrillation (AF) is a common phenomenon of sustained arrhythmia leading to heart failure or stroke. Patients with mental disorders (MD), particularly schizophrenia and bipolar disorder, are at a high risk of AF triggered by the dysregulation of the autonomic nervous system, atrial stretch, oxidative stress, inflammation, and electrical or structural remodeling. Moreover, pathophysiological mechanisms underlying MD may also contribute to the genesis of AF. An overactivated hypothalamic–pituitary–adrenal axis, aberrant renin–angiotensin–aldosterone system, abnormal serotonin signaling, disturbed sleep, and genetic/epigenetic factors can adversely alter atrial electrophysiology and structural substrates, leading to the development of AF. In this review, we provide an update of our collective knowledge of the pathophysiological and molecular mechanisms that link MD and AF. Targeting the pathogenic mechanisms of MD-specific AF may facilitate the development of therapeutics that mitigate AF and cardiovascular mortality in this patient population.

## 1. Introduction

People with mental disorders (MD), particularly schizophrenia and bipolar disorder, are at a 2–4-fold higher risk of mortality than the general population [1, 2, 3], which is attributable not only to suicides and accidents but also to various medical conditions [4, 5]. Large-scale meta-analyses of international studies have unveiled that cardiovascular disease is the primary etiology contributing to the higher mortality due to natural causes in individuals with MD [6, 7].

Atrial fibrillation (AF) was shown to be associated with an elevated risk of stroke and mortality among individuals with MD [8, 9]. National cohort studies from various countries have consistently shown that individuals with MD carry a 1.5–2-fold higher risk of AF than the general population [10, 11, 12, 13]. These findings highlight the urgency of AF as a global public health concern in individuals with MD. Furthermore, given that most cardiovascular risk factors are lifestyle-related [14, 15], the surprising finding that the risk of AF did not vary across individuals with MD from different cultures with different lifestyles implies that some risk factors of AF may be associated with the inherent characteristics of MD in this patient population. Supporting this contention is evidence, gathered from the community and from primary care samples, that the risk of cardiovascular disease in individuals with MD exceeds what can be accounted for by traditional cardiovascular risk factors [16, 17]. Thus, elucidating the MD-specific pathogenic mechanisms of AF may guide the development of therapies aimed at mitigating the risk and mortality rate of AF in individuals with MD.

In this article, we review our current understanding of MD-specific risk factors for AF and the molecular mechanisms mediating the crosstalk between MD and AF.

## 2. Distinctive Pathophysiology and Molecular Mechanisms of AF in Individuals with MD

### 2.1 Hypothalamic–Pituitary–Adrenal Axis

Mounting evidence has implicated the overactivated hypothalamic–pituitary–adrenal axis in the pathophysiology of MD [18]. Cortisol hypersecretion can induce hippocampal and cortical damage through glutamate-induced calcium-dependent excitotoxicity [19], thereby exacerbating psychotic and affective symptoms [20, 21]. Plasma cortisol levels were shown to be positively associated with the risk of AF [22], but the underlying pathogenic mechanisms remain obscure. The mechanism may involve cortisol’s effect on cardiac intracellular calcium homeostasis [23]. Sarcoplasmic reticulum (SR) isolated from the cardiomyocytes of adrenalectomized rats exhibited significantly decreased rates of adenosine triphosphate (ATP)-driven calcium uptake. This was corrected by treatment with exogenous dexamethasone [24]. Furthermore, the administration of hydrocortisone in guinea pigs’ hearts acutely affected cardiac excitation–contraction coupling through protein kinase C (PKC)-dependent shortening of the action potential duration and reduction of the calcium transient amplitudes [25]. The dysregulated calcium homeostasis in atrial myocytes may enhance triggered activity, leading to ectopic atrial activity and occurrence of AF.

### 2.2 Renin–Angiotensin–Aldosterone System

Evidence has suggested the involvement of the renin–angiotensin–aldosterone system in the pathophysiology of MD [26, 27]. The aberrant activation of the renin–angiotensin–aldosterone system is closely linked to the development of AF through multifactorial mechanisms involving adverse cardiac remodeling [28, 29, 30]. Angiotensin II, an essential molecule in the renin–angiotensin–aldosterone system, was shown to bind to angiotensin II receptor type 1 to inhibit nicotinamide adenine dinucleotide phosphate oxidase and elevate the production of reactive oxygen species (ROS) and the activities of proinflammatory transcription factors, such as nuclear factor-kappa B (NF-κB) and E26 transformation-specific sequence-1 [30, 31]. The activation of this signaling pathway subsequently stimulates the release of profibrotic cytokines, such as transforming growth factor-β (TGF-β), tumor necrosis factor-α (TNF-α), interleukin-6 (IL-6), and monocyte chemoattractant protein-1, thereby causing cardiac fibrosis and AF [30, 32]. Moreover, angiotensin II can induce electrophysiological remodeling and AF through the shortening of the effective refractory period and action potential duration in atrial myocytes, in turn potentiating the slow component of delayed rectifier potassium channels [30, 33].

### 2.3 Autonomic Nervous Dysregulation

Autonomic dysregulation is evident in individuals with MD and is associated with different affective states and psychotic symptoms [34, 35]. A dysregulated autonomic nervous system predisposes individuals with MD to greater illness severity and higher cardiometabolic risk [34, 36]. The dysregulation of the autonomic nervous system in individuals with MD is characterized by the predominance of the sympathetic nervous system, either in the form of increased sympathetic tone or decreased parasympathetic activity [36].

Notably, the imbalance of the autonomic nervous system is a highly crucial pathophysiological mechanism underlying the genesis and maintenance of AF [37, 38]. Sympathetic nerve cells release norepinephrine, which binds to β-adrenergic receptors, G protein-coupled receptors, and activate adenylyl cyclase. Activated adenylyl cyclase generates 3^′^,5^′^-cyclic adenosine monophosphate (cAMP), which activates protein kinase A (PKA). PKA subsequently activates downstream calcium handling proteins and ion channels, such as L-type calcium channels at the plasma membrane and the ryanodine receptor 2 (RYR2) at the SR membrane. Activation of these channels induces calcium overload, leading to delayed afterdepolarization. Moreover, the increase in intracellular calcium levels can sustain the activity of calcium/calmodulin-dependent protein kinase II and further amplify the aberrance in calcium handling [38, 39].

Compelling evidence has demonstrated that the overstimulation of β-adrenergic signaling drives the progression of pathological cardiac hypertrophy, ultimately resulting in cardiomyocyte apoptosis and fibrogenesis [40, 41]. In turn, atrial fibrosis further induces re-entry of the electrical current by increasing the heterogeneity of conduction [40, 42].

### 2.4 Abnormal Serotonin Signaling

Serotonin is a key neurotransmitter involved in the pathophysiology of MD [43]. In addition to its activities in the brain, serotonin affects heart function [44]. Elevated levels of serotonin are associated with the risk of coronary artery disease, myocardial hypertrophy, cardiac fibrosis, and arrhythmia [45]. The effects of serotonin are mediated by 5-hydroxy-tryptamine (5-HT) receptors, a group of G protein-coupled receptors widely distributed in the myocardium [46].

The pathogenic mechanisms underlying serotonin-induced AF can be attributed to both the structural and electrophysiological remodeling of the atrium. The 5-HT4 receptor is the main subtype receptor located in the sinoatrial node and pulmonary vein [46]. The expression of mRNAs encoding the 5-HT4 receptor was found to be significantly decreased in the atrial tissues of patients with AF [47, 48]. Through the activation of cAMP, serotonin elevates the L-type calcium current and prolongs action potentials in the atrium [44]. The attenuation of these effects of serotonin is associated with AF [49]. Furthermore, serotonin can induce cardiac fibrosis and pulmonary hypertension through 5-HT2A/2B receptors, leading to adverse structural remodeling and AF [44, 45].

### 2.5 Inflammation

Neuroinflammation is another salient pathophysiological mechanism underlying the development of MD [50, 51, 52]. Activated microglia in the central nervous system secrete proinflammatory cytokines, leading to the dysregulation of neurotransmitters and neurocircuits related to affective and cognitive processing [53, 54, 55]. TNF-α, IL-6, and IL-1β are some of the most prominent cytokines involved in the pathogenesis of MD [50, 51, 52]. Elevated levels of these cytokines have been recorded in cases of first-episode, chronic, and treatment-resistant psychosis [50, 51], as well as mania and depression [56]. Notably, TNF-α, IL-6, and IL-1β are also crucial cytokines implicated in the electrophysiological and structural remodeling associated with AF [57, 58].

Ion channels in cardiomyocytes mediate the effects of inflammatory cytokines that lead to atrial arrhythmogenesis. *In vitro* studies have shown that TNF-α, IL-6, and IL-1β directly act on rat cardiomyocytes to downregulate SR Ca^2+^ ATPase [59, 60, 61, 62], resulting in abnormal calcium handling and increased susceptibility to delayed afterdepolarization. Furthermore, TNF-α, IL-6, and IL-1β suppress L-type calcium currents and calcium transients in cardiomyocytes through a mechanism involving PKC and a nitric oxide-dependent pathway [59, 63, 64], thereby contributing to the reduction of action potential duration and elevating the risk of AF. Gap junction channels, such as connexin40 and connexin43, were also reported to be involved in the inflammatory pathogenesis of AF [58]. The detrimental effects of TNF-α, IL-6, and IL-1β on connexin remodeling have been illustrated using numerous *in vitro* and *in vivo* models [65, 66, 67, 68, 69]. Given the crucial role connexins play in cell-to-cell coupling, their aberrant expression and distribution can profoundly influence the electrical conduction of the atrium.

In addition to doing so through modulation on ion channels and gap junction proteins, TNF-α, IL-6, and IL-1β can lead to the development of AF through the regulation of NF-κB and Nod-like receptor family pyrin domain containing 3 (NLRP3) inflammasome. Studies have demonstrated that TNF-α, IL-6, and IL-1β can activate the NLRP3 inflammasome in cardiomyocytes, which upregulates arrhythmogenic pathways and elevates the levels of NF-κB, ROS, and calcium/calmodulin-dependent protein kinase II [57, 58]. The activation of these arrhythmogenic pathways eventually results in shorter effective refractory periods, fibrotic structural remodeling, and abnormal calcium handling, including remodeling of the RYR2 channel, delayed afterdepolarization, and triggered action potential. Such effects further lead to ectopic activity and re-entry, inducing the central mechanisms underlying AF.

### 2.6 Mitochondrial Dysfunction and Oxidative Stress

The principal function of mitochondria is the production of ATP from various energy substrates. Mitochondrial dysfunction impairs energy metabolism and causes ATP deficiency, thereby perturbing cell function. Furthermore, dysfunctional mitochondria generate large amounts of ROS and disrupt calcium homeostasis, which further damages the mitochondria and lowers ATP levels [70]. Given that neurons and cardiomyocytes are two cell types with relatively high energy demands [71, 72], mitochondrial dysfunction plays a central role in the pathogenesis of both MD and AF [73, 74].

The mitochondrial electron transport chain comprises a series of protein complexes (complex I–V) responsible for the production of ATP [75]. Multiple studies have indicated that individuals with MD exhibit mitochondrial complex dysfunction [76, 77]. Similarly, an analysis of atrial samples from individuals with AF revealed an impairment of the mitochondrial electron transport chain, including reduced activity of complex I and II and increased activity of complex V [78]. Defects in the electron transport chain interfere with ATP synthesis. A meta-analysis of studies analyzing brain samples from individuals with MD revealed that ATP synthesis through the oxidative phosphorylation pathway was attenuated [79]. Similarly, atrial biopsies from individuals with AF revealed aberrant ATP levels [80]. Low ATP levels disrupt intracellular ionic equilibrium and homeostasis by activating cytoplasmic glycolytic enzymes and upregulating lactate synthesis [81]. Lactic acidosis triggers sodium influx and further causes intracellular calcium overload by activating the reverse model of the sodium-calcium exchanger in atrial myocytes, eventually leading to the development of arrhythmogenesis [82, 83].

ROS resulting from mitochondrial dysfunction in atrial cardiomyocytes also exhibit proarrhythmic activities. Markers of mitochondrial oxidative stress were shown to be upregulated in the atria of individuals with AF [78]. Elevated oxidative stress can contribute to the onset of AF by inducing electrophysiological remodeling [84, 85]. Mitochondrial oxidative stress and AF are linked by pathways involving the disrupted function or expression of surface membrane ion channels in atrial myocytes. For instance, ROS can disrupt Nav1.5 function by enhancing late sodium current, which prolongs membrane repolarization and induces early afterdepolarization through the reactivation of voltage-gated calcium ion channels [86]. In addition, ROS can suppress voltage-gated potassium currents either by downregulating the associated ion channels or by altering the phosphorylation of those ion channels through the activities of PKA and PKC. The decrease in voltage-gated potassium currents further prolongs action potentials and results in early afterdepolarization [87]. Furthermore, mitochondrial ROS can downregulate and disarrange connexin40, resulting in a reduction of electrical conductance [88].

Disrupted calcium homeostasis in the mitochondria also contributes to the initiation and progression of AF. Multiple studies have demonstrated that mitochondrial calcium overload can overactivate the Krebs cycle and the electron transport chain, leading to electron leakage and ROS generation. The generated ROS inhibits mitochondrial calcium uptake by causing the opening of mitochondrial permeability transition pores, thereby releasing calcium from the mitochondria and increasing cytosolic calcium concentrations [73, 87, 89]. Furthermore, mitochondrial calcium overload-induced ROS can trigger RYR2 calcium sparks and inhibit SR Ca^2+^ ATPase, resulting in an accentuated calcium efflux from the SR [73, 87]. Collectively, the elevated cytoplasmic calcium levels cause delayed and early afterdepolarization through the activation of the sodium–calcium exchanger and the inhibition of Nav1.5, respectively [73, 87, 89].

In addition to their deleterious effects on electrophysiological remodeling, ROS and calcium overload can promote the development of AF through structural remodeling, which often involves cardiac fibrosis. The senescence of cardiac fibroblasts triggers fibrogenesis in the myocardium [90, 91]. *In vitro* research has illustrated that the most striking features of senescent cardiac fibroblasts are mitochondrial oxidative stress and disrupted calcium homeostasis [92]. The excessive oxidative stress and calcium overload in cardiac fibroblasts further stimulate the release of profibrotic cytokines, such as IL-6 and TGF-β, leading to cardiac fibrogenesis [93, 94]. These *in vitro* findings have been reproduced in transgenic mice exhibiting mitochondria-specific overexpression of nicotinamide adenine dinucleotide phosphate oxidase 4. The mice displayed extensive fibrosis along with elevated TGF-β levels in their myocardium [95].

### 2.7 Sleep Disturbance

Disturbed sleep is an essential component of MD [96, 97] and not only leads to the onset and endurance of the psychological symptoms of MD but also elevates the risk of AF in the patient [98, 99]. The underlying mechanisms are multifactorial and involve the aforementioned pathways and pathophysiological processes, including autonomic nervous dysregulation, systemic inflammation, oxidative stress, overactivation of stress hormones, and the renin–angiotensin–aldosterone system [98, 100]. Moreover, experiments on rapid eye movement sleep-deprived rats have shown that sleep deprivation leads to manic-like behaviors and poor heart function along with upregulation of proarrhythmic signaling molecules, including TGF-β, phosphorylated Smad 2/3, alpha-smooth muscle actin, and store-operated calcium entry channels [101]. These findings suggest that disturbed sleep contributes to the pathogenesis of both MD and AF.

Patients with MD have a high prevalence (approximately 25%) of obstructive sleep apnea (OSA) [102]. Among the different categories of MD diagnoses, higher frequencies of OSA were seen in schizophrenia, major depressive disorder, and posttraumatic stress disorder [102, 103]. The strong association between MD and OSA may involve multiple mechanistic pathways. One principal pathway is mediated by obesity, which is known to be prevalent in people with MD and is a major risk factor for OSA [102, 103]. The elevated obesity risk in patients with MD can be attributed to the metabolic effects of psychotropic medications and several lifestyle risk factors, such as alcohol consumption and cigarette smoking [103]. Sympathetic hyperactivity, neurotransmitter imbalance, overactivation of stress hormones, inflammation, and oxidative stress additionally serve as a common pathophysiology driving the co-evolution of OSA in MD [103]. The hypoxia-hypercapnia resulted from OSA elicits sympathetic overactivation, systemic inflammation, and oxidative stress, in turn causing atrial fibrosis and reduced connexin43 expression [104, 105]. Negative intrathoracic pressure during upper airway obstruction in OSA activates intrathoracic baroreceptors leading to the refractory period shortening promoting AF trigger and perpetuation [104, 105]. Accordingly, both structural and electrophysiological remodeling of the atrium caused by OSA promotes the genesis of AF in individuals with MD.

### 2.8 Genetic and Epigenetic Factors

Large-scale genome-wide association studies have uncovered associations between polygenic risk scores of MD and cardiovascular diseases, including lower heart rate variability, arrhythmia, and diastolic dysfunction [106, 107]. The findings suggest that a genetic predisposition for MD may contribute to the genesis of AF. As shown in Table 1 (Ref. [48, 108, 109, 110, 111, 112, 113, 114, 115, 116, 117, 118, 119, 120, 121, 122, 123, 124, 125, 126, 127, 128, 129, 130, 131, 132, 133, 134, 135, 136, 137, 138, 139, 140, 141, 142, 143, 144, 145, 146, 147, 148, 149, 150, 151, 152, 153, 154]), several overlapping genes were identified in both MD and AF. Considerable genetic overlaps were observed in neurohormones, inflammation, oxidative stress, calcium signaling, and cell membrane ion channels. Hence, the inherited defects in ion channels (ion channelopathies) that regulate both neuronal and cardiac excitability may represent the major genetic susceptibility shared between MD and AF. Genome-wide association studies on ancestrally diverse populations have repeatedly identified calcium voltage-gated channel auxiliary subunit beta 2 (*CACNB2*) as the risk loci associated with MDs such as schizophrenia and bipolar disorder [108, 109, 110, 111]. Additionally, mutations in *CACNB2* are associated with the risk of AF [112]. Future studies should identify *CACNB2* variants in individuals with both MD and AF. MD was also shown to be associated with potassium channels implicated in the pathogenic mechanisms of AF [113, 114, 115, 116, 117, 118, 119, 120, 121, 122, 123, 124, 125]. In addition, emerging data from a randomized controlled trial have shown that ezogabine, a novel antidepressant that opens potassium voltage-gated channel subfamily Q member 2 and 3 (*KCNQ2/3*) type of potassium channels, significantly improves depressive symptoms and anhedonia [155]. Taken together, the literature suggests that hereditary channelopathies may contribute to the pathogenesis of AF in MD.

**Table 1.** **Shared genetic variants in atrial fibrillation (AF) and mental disorder (MD)**.

Protein	Gene	Mechanism of AF	Reference
Ion channel			
Voltage-gated calcium channel	*CACNA2D4*	↓ I_Ca-L_	[112, 113, 115, 126]
	*CACNB2*	↓ I_Ca-L_	[108, 109, 110, 111, 112, 113, 115, 127]
Voltage-gated sodium channel	*SCN5A*	↑ late I_Na_	[113, 115, 128, 129]
		↑ Fibrosis	
	*SCN8A*	↑ late I_Na_	[130, 131]
Voltage-gated potassium channel	*KCNE1*	↑ I_Ks_	[113, 115, 121, 122]
	*KCNE2*	↑ I_Kr_	[113, 115, 119, 121]
	*KCNE4*	↑ I_Ks_	[113, 115, 118, 120]
	*KCNQ1*	↑ I_Ks_	[113, 114, 115, 116]
Inward rectifier potassium channel	*KCNJ3*	↓ I_KAch_	[115, 124, 125]
hyperpolarization activated cyclic nucleotide gated potassium channel	*HCN4*	↑ I_Kf_	[113, 115, 117, 123]
Connexin	*GJA1*	↓ Atrial conduction	[115, 132, 133]
		↑ Fibrosis	
	*GJA5*	↓ Atrial conduction	[115, 134]
		↑ Fibrosis	
Neurohormone	*ACE*	↑ I_Ks_	[135, 136, 137]
		↑ Senescence	
		↑ Fibrosis	
	*ADRB1*	↑ I_Ca-L_	[138, 139, 140]
		↑ RYR2	
		↑ Fibrosis	
	*HTR4*	↑ I_Ca-L_	[48, 141, 142]
		↑ Fibrosis	
Inflammation	*IL1B*	↓ I_Ca-L_	[143, 144, 145]
		↓ SERCA	
		↓ Atrial conduction	
		↑ Fibrosis	
	*IL6R*	↓ I_Ca-L_	[146, 147, 148]
		↓ SERCA	
		↓ Atrial conduction	
		↑ Fibrosis	
	*TNFSF13*	↓ I_Ca-L_	[149, 150]
		↓ SERCA	
		↓ Atrial conduction	
		↑ Fibrosis	
Oxidative stress	*GPX1*	↑ RYR2	[151, 152]
		↓ SERCA	
		↑ Fibrosis	
	*SOD2*	↑ RYR2	[151, 153]
		↓ SERCA	
		↑ Fibrosis	
Calcium signaling	*CAMK2D*	↑ RYR2	[153, 154]

Abbreviation: ↑, upregulation; ↓, downregulation; I_Ca-L_, L-type calcium current; I_KAch_, acetylcholine-sensitive potassium current; I_Kf_, pacemaker current; I_Kr_, rapid component of delayed rectifier potassium channel current; I_Ks_, slow component of delayed rectifier potassium channel current; late I_Na_, late sodium channel current; SERCA, sarco/endoplasmic reticulum Ca^2+^-ATPase; RYR2, ryanodine receptor 2; *CACNA2D4*, calcium voltage-gated channel auxiliary subunit alpha2delta 4; *CACNB2*, calcium voltage-gated channel auxiliary subunit beta 2; *SCN5A*, sodium voltage-gated channel alpha subunit 5; *SCN8A*, sodium voltage-gated channel alpha subunit 8; *KCNE1*, potassium voltage-gated channel subfamily E regulatory subunit 1; *KCNE2*, potassium voltage-gated channel subfamily E regulatory subunit 2; *KCNE4*, potassium voltage-gated channel subfamily E regulatory subunit 4; *KCNQ1*, potassium voltage-gated channel subfamily Q member 1; *KCNJ3*, potassium inwardly rectifying channel subfamily J member 3; *HCN4*, hyperpolarization activated cyclic nucleotide gated potassium channel 4; *GJA1*, gap junction protein alpha 1; *GJA5*, gap junction protein alpha 5; *ACE*, angiotensin I converting enzyme; *ADRB1*, adrenoceptor beta 1; *HTR4*, 5-hydroxytryptamine receptor 4; *IL1B*, interleukin 1 beta; *IL6R*, interleukin 6 receptor; *TNFSF13*, TNF superfamily member 13; *GPX1*, glutathione peroxidase 1; *SOD2*, superoxide dismutase 2; *CAMK2D*: calcium/calmodulin-dependent protein kinase II delta.

**Fig. 1.**
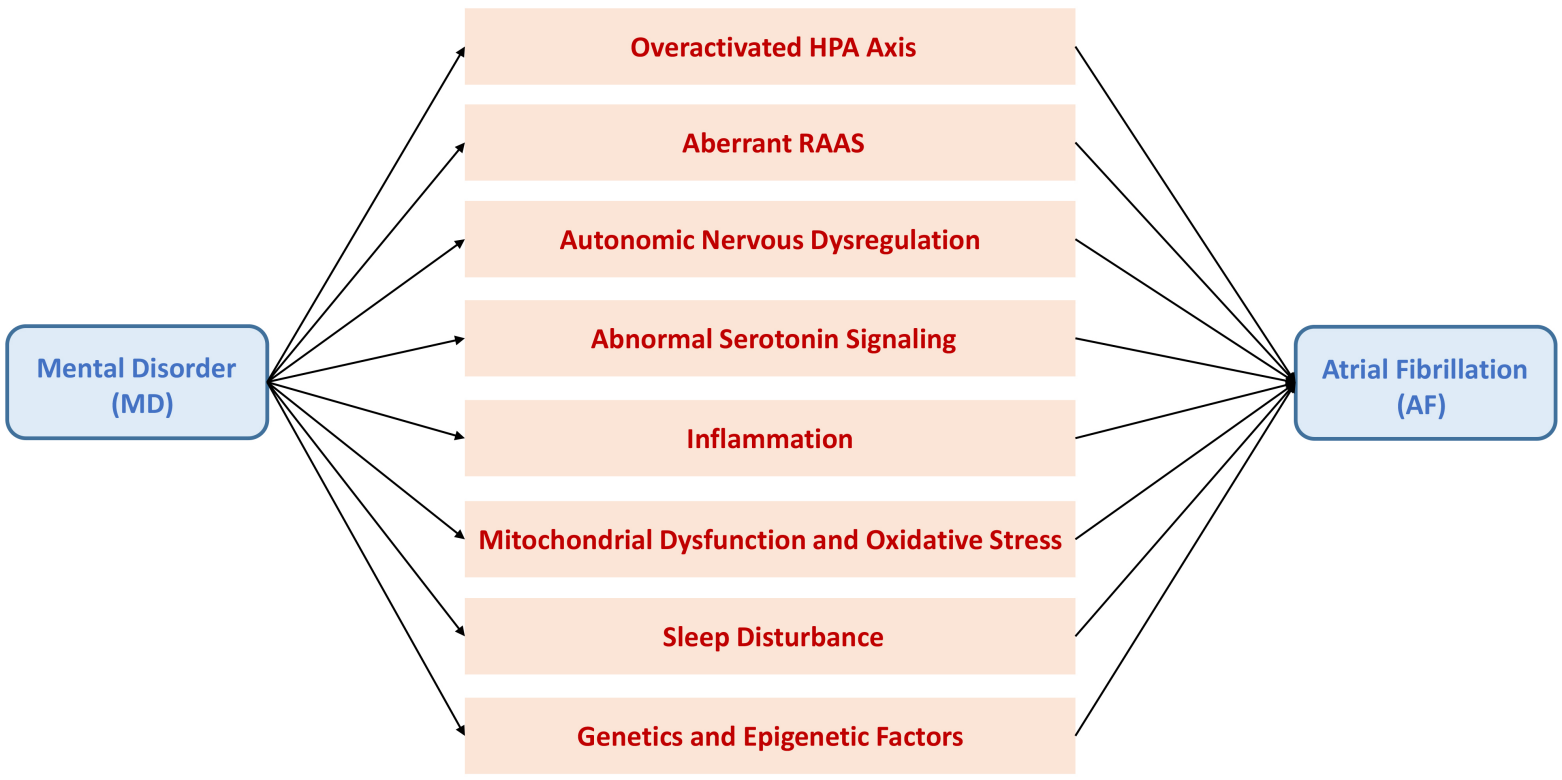
**Putative mechanistic pathways leading to atrial fibrillation (AF) in patients with mental disorder (MD)**. Patients with MD are prone to developing AF through mechanisms involving overactivation of the hypothalamic–pituitary–adrenal (HPA) axis, aberrations in the renin–angiotensin–aldosterone system (RAAS), autonomic nervous dysregulation, abnormal serotonin signaling, inflammation, mitochondrial dysfunction, oxidative stress, sleep disturbance; and genetic and epigenetic factors.

MicroRNAs (miRNAs) belong to a class of endogenous, small noncoding RNAs consisting of approximately 22 nucleotides. By binding to 3^′^-untranslated regions, miRNAs regulate target mRNA and protein expression at the post-transcriptional level [156]. The pathophysiological roles of miRNAs have been well documented in a wide range of human diseases, including MD and AF [157, 158, 159, 160]. A list of common miRNAs associated with both MD and AF is presented in Table 2 (Ref. [157, 159, 161, 162, 163, 164, 165, 166, 167, 168, 169, 170, 171]), along with their target genes and mechanisms. Among them, the miR-34 family, particularly miR-34a, has been intensively investigated because of its central role in the regulation of mitochondrial metabolism, inflammation, senescence, apoptosis, and ion channel function across neurons and cardiomyocytes. MiR-34a was shown to modulate mitochondrial metabolism by targeting genes such as *PPARG* and *ACSL1* [166]. Additionally, miR-34a activates TNF-α, IL-6, IL-1β, and NF-κB signaling by downregulating phosphatase and tensin homolog (*PTEN*) and sirtuin 1 (*SIRT1*) [167]. The downregulation of *SIRT1* and *KL* by miR-34a is closely related to cell senescence [172]. Collectively, these pathways can stimulate atrial fibrosis and subsequent AF in patients with MD. *ANK2* is among the target genes of miR-34a [157]. Ankyrin 2 (*ANK2*) encodes ankyrin-B, a major scaffolding protein responsible for the localization and spatial organization of ion channels, transporters, structural proteins, and signaling molecules involved in a wide range of fundamental cellular processes [172]. The suppression of *ANK2* expression by miR-34a induces electrophysiological and structural remodeling, resulting in the onset of AF [172]. Fig. 1 summarizes the putative mechanistic pathways leading to AF in patients with MD.

**Table 2.** **Shared dysregulated microRNAs in atrial fibrillation (AF) and mental disorder (MD)**.

MicroRNA	Expression	Target gene	Mechanism	Reference
miR-21	AF: ↑↓	*CACNA1C*, *CACNB2*, *PTEN*, *SMAD7*, *STAT3*	↓ I_Ca-L_	[157, 159, 161, 162]
	MD: ↑		↑ Inflammation	
			↑ Oxidative stress	
			↑ Mitochondrial dysfunction	
			↑ Fibrosis	
miR-24	AF: ↑	*NOS3*	↑ Oxidative stress	[157, 163]
	MD: ↓		↑ Mitochondrial dysfunction	
			↑ Fibrosis	
miR-27	AF: ↑	*TGFBR1*, *GJA5*	↓ Atrial conduction	[157, 162, 163]
	MD: ↑		↑ Fibrosis	
miR-29	AF: ↓	*COL1A1*, *COL3A1*, *FBN*	↑ Fibrosis	[157, 162, 163]
	MD: ↑↓			
miR-30	AF: ↓	*KCNJ3*, *KCNJ5*, *SNAI1*	↓ I_KAch_	[157, 159, 162, 163, 164]
	MD: ↑↓		↑ Fibrosis	
miR-31	AF: ↑	*CACNA1C*, *KCNJ3*, *NOS1*	↓ I_Ca-L_	[157, 159, 165]
	MD: ↓		↓ I_KAch_	
			↑ Inflammation	
			↑ Oxidative stress	
			↑ Mitochondrial dysfunction	
			↑ Fibrosis	
miR-34	AF: ↑	*ACSL1*, *ANK2*, *KL*, *PPARG*, *PTEN*, *SIRT1*, *SMAD4*	↑ Inflammation	[157, 159, 162, 163, 166, 167, 168, 169]
	MD: ↑		↑ Oxidative stress	
			↑ Mitochondrial dysfunction	
			↑ Senescence	
			↑ Fibrosis	
miR-106	AF: ↓	*RYR2*	↑ SR calcium leak	[157, 162, 168]
	MD: ↑			
miR-432	AF: ↓	*ACE*, *CDKN2B*, *SMAD2*	↑ Inflammation	[157, 159, 168]
	MD: ↓		↑ Oxidative stress	
			↑ Senescence	
			↑ Fibrosis	
miR-499	AF: ↑	*CACNB2*, *KCNN3*	↓ I_Ca-L_	[162, 169, 170, 171]
	MD: ↓		↓ I_SK_	

Abbreviation: ↑, upregulation; ↓, downregulation; I_Ca-L_, L-type calcium current; I_KAch_, acetylcholine-sensitive potassium current; I_SK_, small-conductance calcium-activated potassium channel current; SR, sarcoplasmic reticulum; *CACNA1C*, calcium voltage-gated channel subunit alpha1 C; *CACNB2*, calcium voltage-gated channel auxiliary subunit beta 2; *PTEN*, phosphatase and tensin homolog; *SMAD7*, SMAD family member 7; *STAT3*, signal transducer and activator of transcription 3; *NOS3*, nitric oxide synthase 3; *TGFBR1*, transforming growth factor beta receptor 1; *GJA5*, gap junction protein alpha 5; *COL1A1*, collagen type I alpha 1 chain; *COL3A1*, collagen type III alpha 1 chain; *FBN*, fibrillin; *KCNJ3*, potassium inwardly rectifying channel subfamily J member 3; *KCNJ5*, potassium inwardly rectifying channel subfamily J member 5; *SNAI1*, snail family transcriptional repressor 1; *NOS1*, nitric oxide synthase 1; *ACSL1*, acyl-CoA synthetase long chain family member 1; *ANK2*, ankyrin 2; *KL*, klotho; *PPARG*, peroxisome proliferator activated receptor gamma; *SIRT1*, sirtuin 1; *SMAD4*, SMAD family member 4; *RYR2*, ryanodine receptor 2; *ACE*, angiotensin I converting enzyme *CDKN2B*, cyclin dependent kinase inhibitor 2B; *SMAD2*, SMAD family member 2, *KCNN3*, potassium calcium-activated channel subfamily N member 3.

## 3. Other Aggravating Factors for AF in Individuals with MD

### 3.1 Delayed Intervention for AF Prevention

**Fig. 2.**
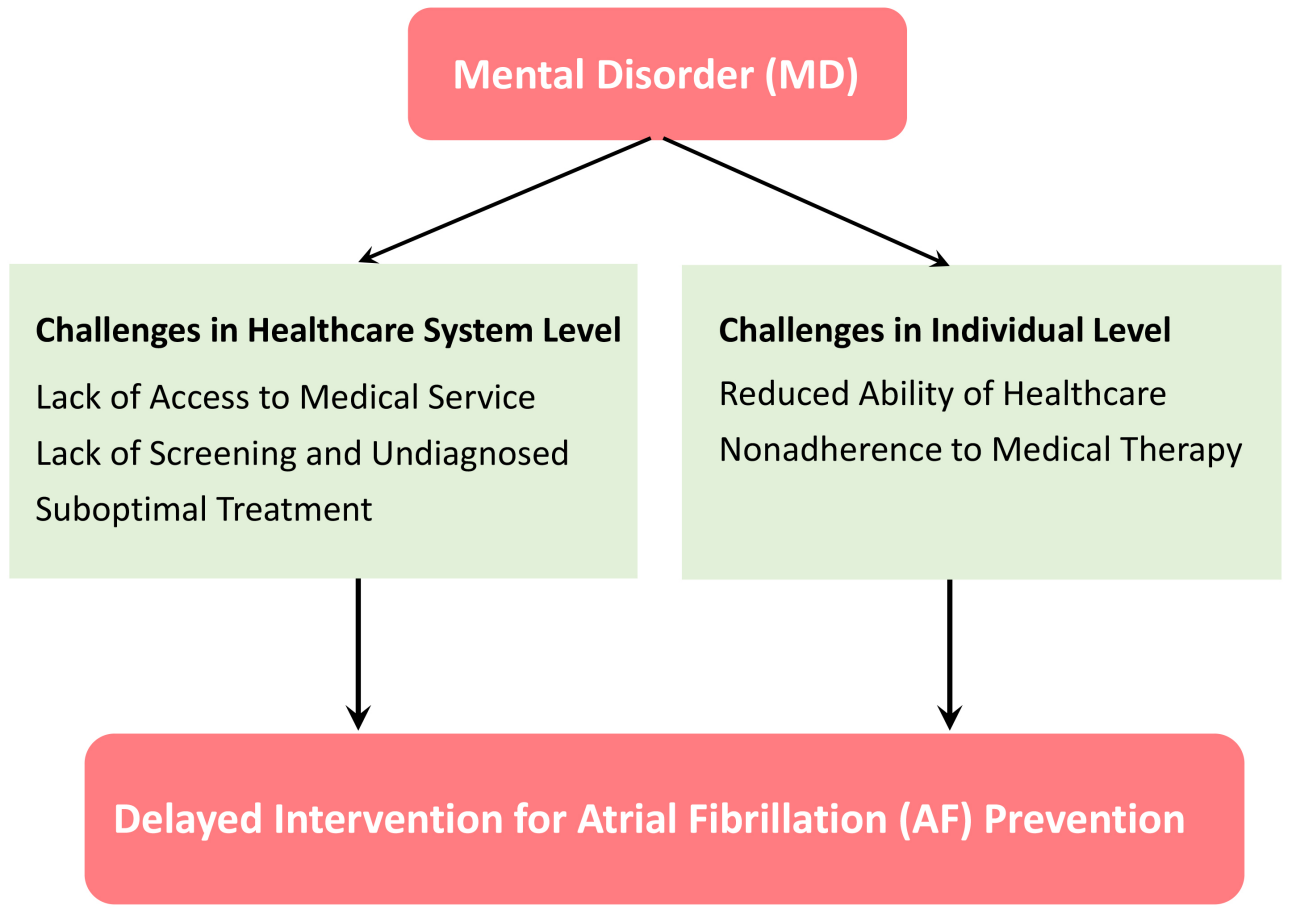
**Challenges leading to the delayed intervention for atrial fibrillation (AF) prevention in patients with mental disorder (MD)**. Healthcare system factors include the disparities in access to medical service, lack of screening and diagnosis, and suboptimal treatment for risk factors of AF. Patient factors encompass the reduced ability to maintain healthcare and nonadherence to medical therapies aimed at modifying risk factors of AF.

Increasing evidence indicates that greater burdens of psychotic and affective symptoms are associated with a higher cardiovascular risk in individuals with MD [173, 174, 175, 176]. One candidate factor linking psychological symptoms and cardiovascular risk in individuals with MD is the reduced ability of healthcare. For instance, adherence to antipsychotic medication and to medicines for hypertension, hyperglycemia, and hyperlipidemia is difficult for patients with MD [177, 178]. This poor medication adherence is more pronounced in patients with more severe psychopathology conditions [179], who are therefore more likely to develop cardiovascular diseases because of inadequate treatment of cardiovascular risk factors. Furthermore, numerous studies have reported marked disparities in the identification and treatment of cardiovascular diseases among individuals with MD [180, 181, 182]. The lack of access to optimal care for cardiovascular diseases in the early stage can exacerbate the severity of structural and electrophysiological abnormalities in the myocardium, eventually leading to the development of AF. Fig. 2 summarizes the challenges leading to delayed intervention for AF prevention in patients with MD.

### 3.2 Psychotropic Medication

**Fig. 3.**
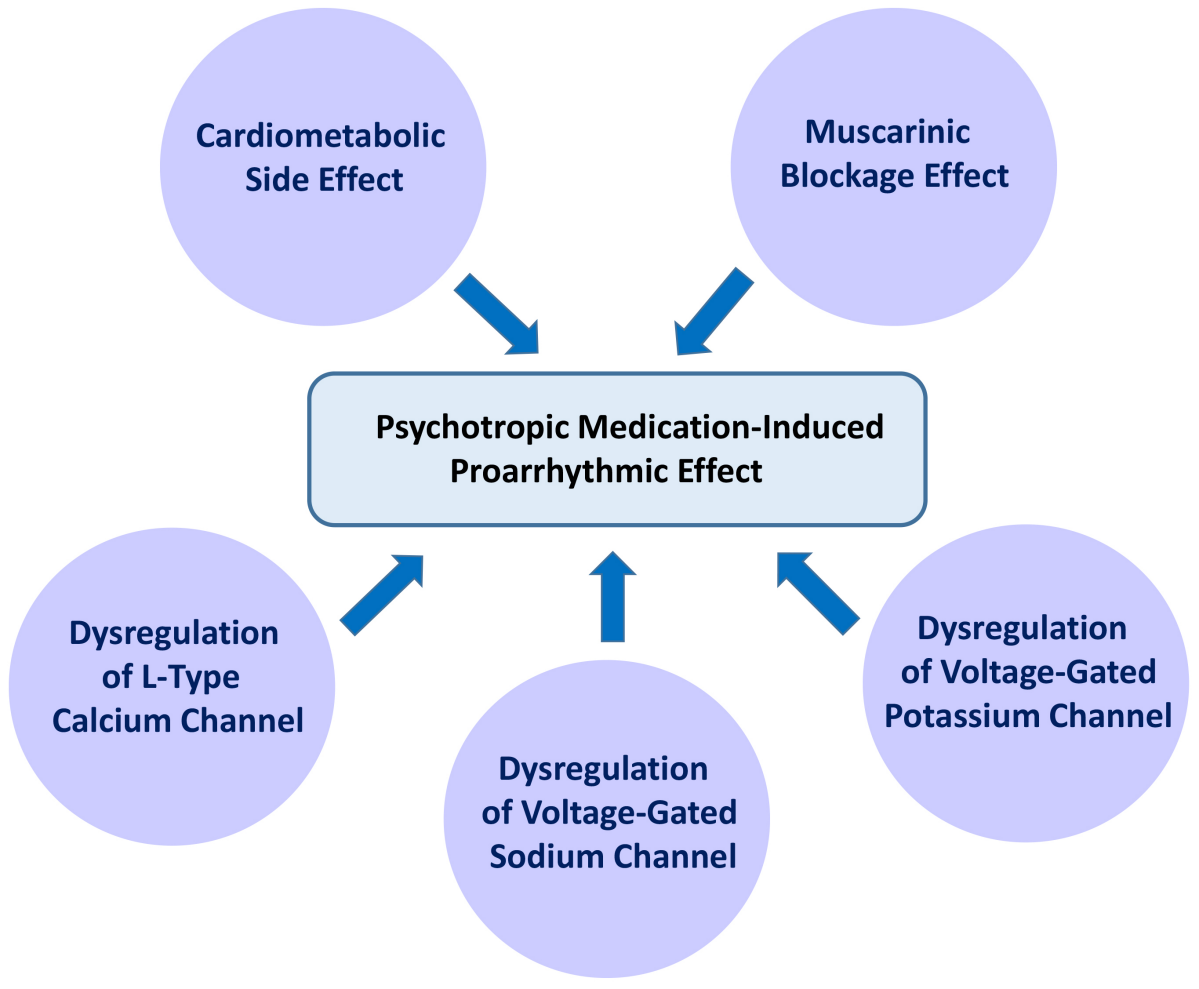
**Pharmacodynamic properties resulting in the proarrhythmic effects of psychotropic medications**. Numerous guidelines recommend psychotropic medications as the first-line therapy to treat mental disorders (MD). However, the cardiometabolic side effects of psychotropic medications, especially the second-generation antipsychotics, heighten the risk of developing coronary artery disease and atrial fibrillation (AF). Furthermore, the muscarinic blockage effects and dysregulation of voltage-gated ion channels caused by psychotropic medications interfere with electrical conduction in the atrium.

The effects of psychotropic medication on the cardiovascular system have been extensively investigated. Fig. 3 summarizes pharmacodynamic properties resulting in the proarrhythmic effects of psychotropic medications. The use of antipsychotics is associated with cardiometabolic side effects that increase the risk of developing coronary artery disease and AF [183]. Moreover, the muscarinic blockage effects of antipsychotics can directly interfere with atrial conduction and elevate the risk of AF in individuals with MD [184]. The proarrhythmic effects of serotonin on L-type calcium currents and action potential duration also contribute to the elevated risk of AF [49, 185]. Conversely, some studies have reported associations between antipsychotic medication and a reduced risk of cardiovascular mortality in individuals with MD [186, 187]. These beneficial effects may result from the alleviation of psychological symptoms and the improvement of healthcare capability [188].

## 4. Challenges and Directions for Future Research

Building on the aforementioned studies, future studies should unravel the pathophysiological pathways leading to AF in individuals with MD, which will aid future translational research and clinical trials aimed at managing AF among individuals with MD. However, some challenges remain. First, the results of mechanistic studies on AF and MD have mostly been obtained from experiments in cell lines and animal models. None of these models can exactly reflect the disease phenotypes of individual patients. An increasing number of studies in clinical and biomedical science have employed induced pluripotent stem cells and organoids derived from such cells to study the molecular mechanisms underlying human diseases. This approach has been increasingly adopted in the fields of cardiology and psychiatry [189, 190] and holds great promise for uncovering the pathogenesis of MD-specific AF. Second, the influence of psychotropic medication and medical comorbidities poses a challenge in the translation of laboratory findings to clinical studies in individuals with MD. This difficulty can be resolved by studying the patients during the early stages of MD when the effects of psychotropic medication and medical comorbidities are not obvious. Remarkably, a study reported that individuals with early-stage MD already exhibit precursors of cardiovascular diseases despite normal gross cardiac structure and function [191]. Such precursors can be incorporated as risk factors of AF specific to patients with early-stage MD in future translational studies.

## 5. Conclusions

Our review suggests that MD and AF may share a common molecular pathophysiology in addition to traditional cardiovascular risk factors. Future studies should uncover the mechanistic roots underlying the development of MD-specific AF. Knowledge of these mechanisms may inform the development of novel therapeutic strategies for mitigating the risk of AF and mortality among individuals with MD.
